# Supplementary material for: From sequence to enzyme mechanism using multi-label machine learning
Source: BMC Bioinformatics. 2014 May 19;15:150. doi: 10.1186/1471-2105-15-150 (PMC4229970; doi:10.1186/1471-2105-15-150)
Supplement: Additional file 2 — Java code of ml2db. Additional file ml2db_code.tar.gz contains the Java source code to run the multi-label machine learning experiments and save the results to database. The code’s Javadoc is included. [file 1471-2105-15-150-S2.zip › additional file 2/ml2db/utils/doc/uk/ac/ed/inf/utils/collectionutils/ListUtils.html]

ListUtils


---


|  |  |  |
| --- | --- | --- |
| **Overview** \| **Package** \| **Class** \| **Use** \| **Tree** \| **Deprecated** \| **Index** \| **Help** | |  |
| **PREV CLASS**   **NEXT CLASS** | **FRAMES**    **NO FRAMES**     **All Classes** |  |
| SUMMARY: NESTED \| FIELD \| CONSTR \| METHOD | DETAIL: FIELD \| CONSTR \| METHOD |  |


---


## uk.ac.ed.inf.utils.collectionutils Class ListUtils

```
java.lang.Object
  uk.ac.ed.inf.utils.collectionutils.ListUtils
```

---

``` public class ListUtils extends java.lang.Object ```

Utilities to manipulate lists

**Version:**
:   4 Jan 2008

**Author:**
:   Luna De Ferrari luna.deferrari-at-ed.ac.uk

---

**Constructor Summary**

`ListUtils()`


| **Method Summary** | |
| --- | --- |
| `static java.lang.String[]` | `addStringToArray(java.lang.String[] array, java.lang.String textToAdd)`             Takes an array and adds a string to all elements |
| `static java.util.ArrayList<java.lang.String>` | `addStringToArrayList(java.util.ArrayList list, java.lang.String textToAdd)`             Takes an arrayList and adds a string to all elements |
| `static java.util.Set<java.lang.String>` | `cleanSet(java.util.Set<java.lang.String> set)`             Cleans a set from null and empty elements |
| `static java.util.LinkedHashSet` | `getCommonElements(java.util.ArrayList list1, java.util.ArrayList list2)`             Gets the elements present in both lists |
| `static int` | `getLongestStringLength(java.util.ArrayList<java.lang.String> array)`             Get the length of the longest string in an array |
| `static int` | `getShortestStringLength(java.util.ArrayList<java.lang.String> array)`             Get the length of the shortest string in an array |
| `static boolean` | `isNullOrEmpty(java.lang.String string)`             tells if a string is null or empty |
| `static boolean` | `isNullOrEmpty(java.util.Vector<java.lang.String> list)`             tells if a list is null or empty |
| `static void` | `printList(java.util.List list)`             Prints an array as a list of 'index' + 'array element' (with a header containing the size) |
| `static void` | `printListsOfLists(java.util.List<java.util.List> list)`             Prints lists of lists |
| `static java.util.TreeSet<java.lang.String>` | `setFromList(java.util.ArrayList<java.lang.String> list)`             create a set (elements appear only once) from a list |
| `static java.util.Vector<java.lang.String>` | `vectorFromList(java.util.ArrayList<java.lang.String> list)` |

**Methods inherited from class java.lang.Object**

`equals, getClass, hashCode, notify, notifyAll, toString, wait, wait, wait`

**Constructor Detail**

### ListUtils

```
public ListUtils()
```


**Method Detail**

### addStringToArray

```
public static java.lang.String[] addStringToArray(java.lang.String[] array,
                                                  java.lang.String textToAdd)
```

:   Takes an array and adds a string to all elements

---


### addStringToArrayList

```
public static java.util.ArrayList<java.lang.String> addStringToArrayList(java.util.ArrayList list,
                                                                         java.lang.String textToAdd)
```

:   Takes an arrayList and adds a string to all elements

---


### cleanSet

```
public static java.util.Set<java.lang.String> cleanSet(java.util.Set<java.lang.String> set)
```

:   Cleans a set from null and empty elements

    :   **Parameters:**: `set` - **Returns:**: a set without the null element or the empty string

---


### getCommonElements

```
public static java.util.LinkedHashSet getCommonElements(java.util.ArrayList list1,
                                                        java.util.ArrayList list2)
```

:   Gets the elements present in both lists

    :   **Parameters:**: `list1` - a list: `list2` - another list **Returns:**: the set containing the common elements (each element only once)

---


### getLongestStringLength

```
public static int getLongestStringLength(java.util.ArrayList<java.lang.String> array)
```

:   Get the length of the longest string in an array

    :   **Parameters:**: `array` - an array of strings **Returns:**: the length of the longest string

---


### getShortestStringLength

```
public static int getShortestStringLength(java.util.ArrayList<java.lang.String> array)
```

:   Get the length of the shortest string in an array

    :   **Parameters:**: `array` - an array of strings **Returns:**: the length of the shortest string

---


### isNullOrEmpty

```
public static boolean isNullOrEmpty(java.lang.String string)
```

:   tells if a string is null or empty

    :   **Parameters:**: `string` - a string **Returns:**: true if the string is null or empty (no characters), false otherwise

---


### isNullOrEmpty

```
public static boolean isNullOrEmpty(java.util.Vector<java.lang.String> list)
```

:   tells if a list is null or empty

    :   **Parameters:**: `list` - **Returns:**: true if the list is null or empty, false otherwise

---


### printList

```
public static void printList(java.util.List list)
```

:   Prints an array as a list of 'index' + 'array element' (with a header
    containing the size)

    :   **Parameters:**: `list` - the List to print

---


### printListsOfLists

```
public static void printListsOfLists(java.util.List<java.util.List> list)
```

:   Prints lists of lists

    :   **Parameters:**: `list` -

---


### setFromList

```
public static java.util.TreeSet<java.lang.String> setFromList(java.util.ArrayList<java.lang.String> list)
```

:   create a set (elements appear only once) from a list

---


### vectorFromList

```
public static java.util.Vector<java.lang.String> vectorFromList(java.util.ArrayList<java.lang.String> list)
```


---


|  |  |  |
| --- | --- | --- |
| **Overview** \| **Package** \| **Class** \| **Use** \| **Tree** \| **Deprecated** \| **Index** \| **Help** | |  |
| **PREV CLASS**   **NEXT CLASS** | **FRAMES**    **NO FRAMES**     **All Classes** |  |
| SUMMARY: NESTED \| FIELD \| CONSTR \| METHOD | DETAIL: FIELD \| CONSTR \| METHOD |  |


---
